# Reconstruction of the coracoclavicular ligament with palmaris longus tendon and Mersilene tape for acromioclavicular dislocations

**DOI:** 10.1186/s12891-022-05589-y

**Published:** 2022-07-06

**Authors:** Yoon-Min Lee, Joo Dong Yeo, Zin Ouk Hwang, Seok-Whan Song, Yoo Joon Sur

**Affiliations:** 1 Department of Orthopedic Surgery, Yeouido St. Mary’s Hospital, The Catholic University of Korea, Seoul, Republic of Korea; 2 Department of Orthopedic Surgery, Uijeongbu St. Mary’s Hospital, The Catholic University of Korea, 271, Cheonbo-ro, Uijeongbu, Gyeonggi-do 11765 Republic of Korea

**Keywords:** Dislocation, Shoulder injury, Acromioclavicular joint, Coracoclavicular ligament, Palmaris longus, Mersilene tape

## Abstract

**Background:**

Acromioclavicular (AC) joint dislocation is common among shoulder injuries, and various surgical methods have been introduced for effective ligament reconstruction. Reconstruction of the coracoclavicular (CC) ligament in the anatomical position using autologous tendons is a recent surgical trend. This study is to report clinical and radiologic results of reconstruction of the CC ligament using an autologous palmaris longus tendon interweaved with Mersilene tape (PLMT) with a minimum 2-year follow-up.

**Methods:**

This retrospective study analyzed 76 patients (mean age, 43.4 ± 11.2 years) with AC joint dislocation treated by reconstruction of the CC ligament with PLMT, from March 2004 to February 2017. The mean follow-up period was 28 ± 6.7 months (range, 24–66 months). The Visual Analog Scale (VAS) for pain assessment, American Shoulder and Elbow Surgeons rating scale (ASES), and Constant Score (CS) were used to evaluate clinical outcomes at the preoperative and the final follow-ups. CC and AC distances were measured using anteroposterior (AP) X-ray preoperatively and at the final follow-up for radiologic outcomes. Complications were also assessed.

**Results:**

The mean preoperative VAS for pain, ASES, CS were 5.7 ± 0.7, 77.1 ± 6.2, and 61.5 ± 5.2, respectively. These scores at the final follow-up improved to 2.1 ± 0.5, 90.9 ± 4.3, and 94 ± 7.0, respectively (*p* = 0.043, *p* <  0.001, *p* <  0.001). The mean preoperative CC and AC distances were 16.49 ± 3.73 mm and 13.84 ± 3.98 mm, respectively. The final follow-up CC and AC distances were 9.29 ± 2.72 mm and 5.30 ± 2.09 mm, respectively (*p* <  0.001, *p* <  0.001). Although a slight re-widening of the CC distance occurred in 10 patients (13.1%), most patients regained full range of motion of the affected shoulder at the final follow-up.

**Conclusion:**

The CC ligament reconstruction with PLMT for the treatment of AC joint dislocation showed good clinical and radiological results. This technique could be a good alternative treatment for AC dislocations.

## Background

Dislocation of the acromioclavicular (AC) joint is a common shoulder injury, with an estimated incidence of 17% among all shoulder injuries [1, 6, 17, 19]. Numerous surgical procedures have been introduced for AC joint dislocation [13, 15, 26], but no single definitive treatment standard has been established to date. In recent studies, the optimal treatment for AC dislocation has been the restoration of static stability of the coracoclavicular (CC) ligament; which resists against the repetitive axial and rotational motion of the clavicle [6]. Thus, anatomical reconstruction with free tendon grafts or artificial material has recently received increasing interest, with reports of improved stability of the joint with good clinical results [2]. According to several comparative studies and systematic reviews, reconstruction of the CC ligament by free tendon graft can provide the highest subjective scores and the fewest complications with low reoperation rates [8, 10, 18]. However, these techniques are associated with some complications, such as clavicle fractures caused by bone tunnels or early mechanical failure of grafted material, whether it is an autologous tendon or an artificial material [11, 14, 18, 22].

In this study, we devised a surgical technique using the ipsilateral autologous palmaris longus tendon interweaved with Mersilene tape (Ethicon, Somerville, NJ, USA) (PLMT) to obtain both initial stability from the artificial tape and later stability by ingrowth of biologic cells into the grafted tendon. To avoid complications associated with clavicular bone tunnels, ligament reconstruction was performed using the under-coracoid-around-clavicle pathway [24]. This study aimed to introduce our surgical technique using PLMT and report results of clinical and radiologic outcomes with a minimum 2-year follow-up.

## Methods

### Study design

After approval of the Institutional Review Board (SC18RESI0007), a total of 113 patients with Rockwood classification grade III to V AC joint dislocations, from March 2004 to February 2017, were reviewed retrospectively. For grade III patients, we explained them pros and cons of non-surgical treatment (using Kenny-Howard brace) and surgical treatment, we performed surgery only for those who decided the surgical treatment, and also performed surgery who wanted early return to work or exercises. All methods were performed in accordance with the relevant guidelines and regulations. Patients who had undergone reconstruction of the CC ligament using the PLMT with a follow-up period of over 2 years were included. Patients with minor concomitant shoulder pathologies, such as rotator cuff tear, labral tear, and biceps tendinopathy, and if they had ipsilateral upper extremity injuries such as fractures in the clavicle, scapula, and humerus were excluded. Consequently, among 113 patients, 37 patients were excluded, and 76 patients were finally included in this study. The patients who had undergone operations at 6 weeks after the injury were regarded as chronic cases, but the surgical technique was the same as for the acute cases.

### Surgical techniques

**Fig. 1:**
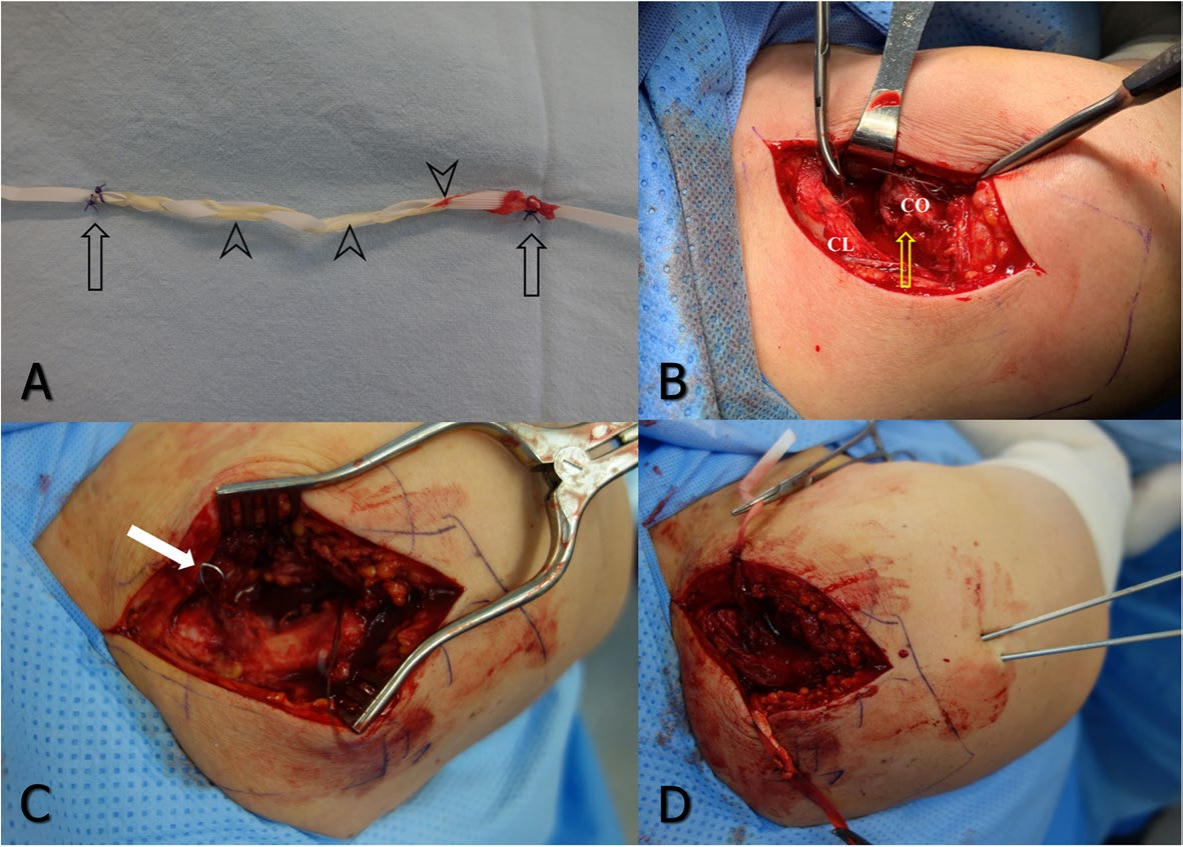
Techniques for PLMT passing under the coracoid process and over the clavicle. **a** A harvested PL tendon (arrow heads) was prepared by interweaving with Mersilene tape. Both ends of the tendon were sutured to the tape so as not to be separated during the procedure (arrows). **b** A 23-gauge roll wire was inserted into the hole of a wire passer, and the passer was inserted under the coracoid process. Ruptured coracoclavicular ligament was visible (arrow). CO: coracoid process, CL: clavicle. **c** The passer was removed leaving the roll wire. **d** The AC joint was reduced and fixed with two S-pins

**Fig. 2:**
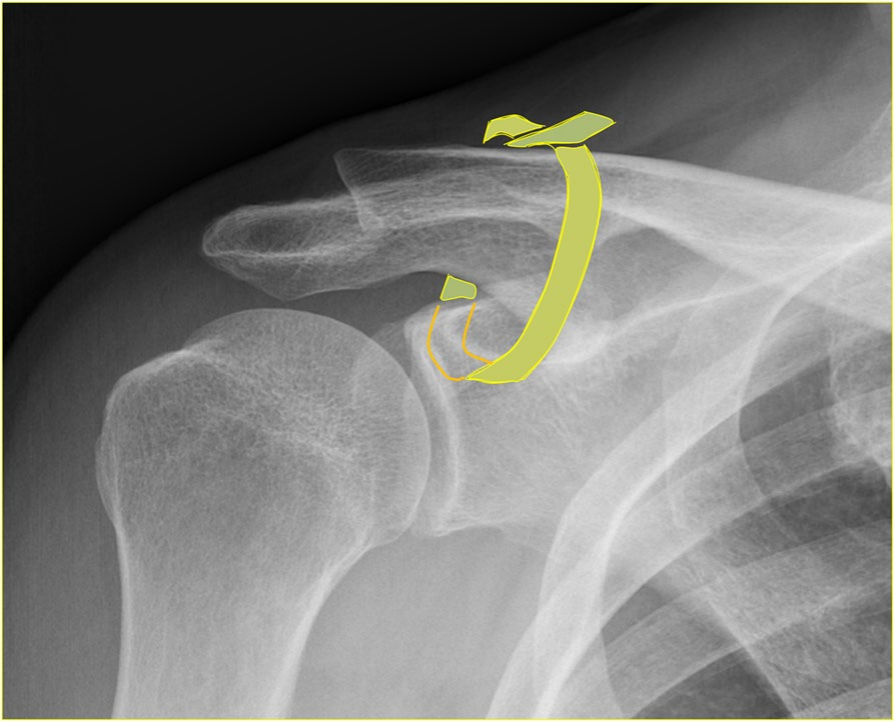
This schematic illustration shows the PLMT reconstruction of the CC ligament

Patients were placed in the beach chair position under general endotracheal anesthesia. A palmaris longus (PL) tendon was harvested from the ipsilateral forearm using a tendon stripper, interweaved with Mersilene tape (Fig. 1A), and wrapped with a saline-soaked gauze. A transverse skin incision, approximately 10 cm in length, was made over the distal clavicle including the AC joint. The deltotrapezial fascia was elevated subperiosteally to expose the clavicle and AC joint. Dissection was performed as minimal as possible to allow passing the PLMT underneath the coracoid process for indirect healing of surrounding soft tissues. The distal clavicle was retracted superoposteriorly to expose the coracoid process and after identification of the coracoid process, a wire passer was passed under the coracoid process with a 23-gauge roll wire (Fig. 1B). The passer was removed, leaving the roll wire under the coracoid process (Fig. 1C), and the PLMT was passed beneath the coracoid process with the passed roll wire. One end of the PLMT then passed posteriorly to encircle the coracoid process and clavicle. Subsequently, the dislocated AC joint was reduced and fixed with two Steinmann pins (S-pins) (Fig. 1D). The PLMT was sutured together in a fully tightened state (Fig. 2). The deltotrapezial fascia was closed securely for additional stability.

### Postoperative care

The injured arm was immobilized for 6 weeks with a Velpeau brace, and the S-pins were removed at 6 weeks after the operation. Pendulum and gentle passive stretching exercises of the shoulder were initiated after removal of S-pins. Patients were allowed to start active range of motion and strengthening exercises 9 weeks after the operation. Return to work and light sport activities were allowed 12 weeks after the operation. If the progression of re-widening was identified after removal of S-pins, we recommend the patient to restrict use of the arm and use the arm sling during daily activities to prevent further widening. However, patients were advised not to perform contact sports for at least 6 months.

### Clinical assessment

We contacted the patients at least 2 years after the surgery (range, 24–66 months) and scheduled them for a clinical examination with the same orthopedic doctor who performed the surgery. Range of motion was checked in the following planes: flexion, abduction, internal and external rotations at 90° of shoulder abduction, and extension. For the clinical outcome assessment, the Visual Analog Scale (VAS) for pain, American Shoulder and Elbow Surgeons rating scale (ASES) score, and Constant Score (CS) were evaluated at the preoperative and the final follow-ups.

### Radiologic assessment

The CC and AC distances were measured using clavicle anteroposterior (AP) radiographs. The CC distance was defined as the distance between the most superior point of the coracoid process and the nearest point of the inferior surface of the clavicle. The AC distance was defined as the shortest distance between the distal clavicle and acromion. The distances were measured preoperatively, at 6 weeks postoperatively, and at the final follow-up. The CC and AC distances of the uninjured shoulder were measured also to compare with the injured shoulder. Since the CC and AC distances could be sufficiently confirmed with simple clavicle X-rays, additional measurements in computed tomography (CT) were not considered.

### Statistical analyses

Statistical analysis was performed with PASW software, ver. 21 (SPSS Inc., Chicago, IL). Paired student’s *t*-test was used to detect differences between preoperative and postoperative outcome scores, pain scales and radiologic measurements. A *P*-value less than .05 was considered as statistically significant.

## Results

The patients comprised 66 men and 10 women, with a mean age of 43.4 ± 11.2 years (range, 16–82 years). The right shoulder was involved in 52 patients, and the left shoulder in 24 patients. According to the Rockwood classification for AC-CC injury, there were 15 cases of type III, 5 cases of type IV, and 56 cases of type V. Sixty-one shoulders (80.2%) were classified as acute injuries, and the mean time interval between the injury and the operation was 16.5 ± 15.3 days (range, 3–35 days). Fifteen shoulders (19.8%) were chronic injuries and were operated at 87.3 ± 11.7 days (range, 65–150 days) after injury on average. Thirty-four patients (44.7%) were injured during contact sports activities, such as soccer, basketball, and martial arts, 17 patients (22.4%) were injured due to traffic accidents, 18 patients (23.8%) were injured while riding a bicycle, 3 patients (3.9%) fell from a height, and 4 patients (5.2%) had fall down injuries. The mean follow-up period was 28 ± 6.7 months (range, 24–66 months) (Table 1).

**Table 1:** Patients’ demographic characteristics

	Data
Male: Female	66:10
Age, y	43.4 ± 11.2 (16–82)
Injured site, n	
Right	52
Left	24
Rockwood classification^a^, n	
III	15
IV	5
V	56
Time from injury to surgery, days	
Acute	16.5 ± 15.3 (3–35)
Chronic	87.3 ± 11.7 (65–150)
Injury mechanism, n	
Sports injury (contact sports)	34
Traffic accident	17
Bicycle accident	18
Fall from height (more than 2 m)	3
Fall down	4
Follow-up, mo	28 ± 6.7 (24–66)

Values are reported as mean ± standard deviation

*n* number, *y* year, *mo* month

^a^Rockwood classification of AC dislocations

### Clinical outcomes

The mean ranges of motion of the shoulder at the final follow-up were 165° ± 15.3° (range, 140–180°) in forward flexion, 146.7° ± 20.1° (range, 110–180°) in lateral abduction, 55.7° ± 11.4° (range, 20–80°) in external rotation, 59.4° ± 16.8° (range, 30–90°) in internal rotation, and 32.5° ± 8.1° (range, 20–45°) in extension. The mean VAS for pain were decreased from 5.7 ± 0.7 points (range, 3–9 points) preoperatively to 2.1 ± 0.5 points (range, 0–5 points) at the final follow-up. The mean ASES score improved from 77.1 ± 6.2 points (range, 65–90 points) to 90.9 ± 4.3 points (range, 77–100 points). The mean CS improved from 61.5 ± 5.2 points (range, 41–68 points) preoperatively to 94 ± 7.0 points (range, 68–95 points) at the final follow-up. All clinical scores showed statistically significant improvements (*p* = 0.043, *p* <  0.001, *p* <  0.001) (Table 2).

**Table 2:** Summary of clinical outcomes

Outcome measurements	Preoperative	Last follow-up	*P* value
VAS	5.7 ± 0.7 (3–9)	2.1 ± 0.5 (0–5)	0.043*
ASES	77.1 ± 6.2 (65–90)	90.9 ± 4.3 (77–100)	< 0.001*
CS	61.5 ± 5.2 (41–68)	94.0 ± 7.0 (68–95)	< 0.001*

Values are reported as mean ± standard deviation

*VAS* Visual Analog Scale, *ASES* American Shoulder and Elbow Surgeons Standardized Shoulder Assessment Form, *CS* Constant Score

*Independent Paired t-test

### Radiologic outcomes

**Fig. 3:**
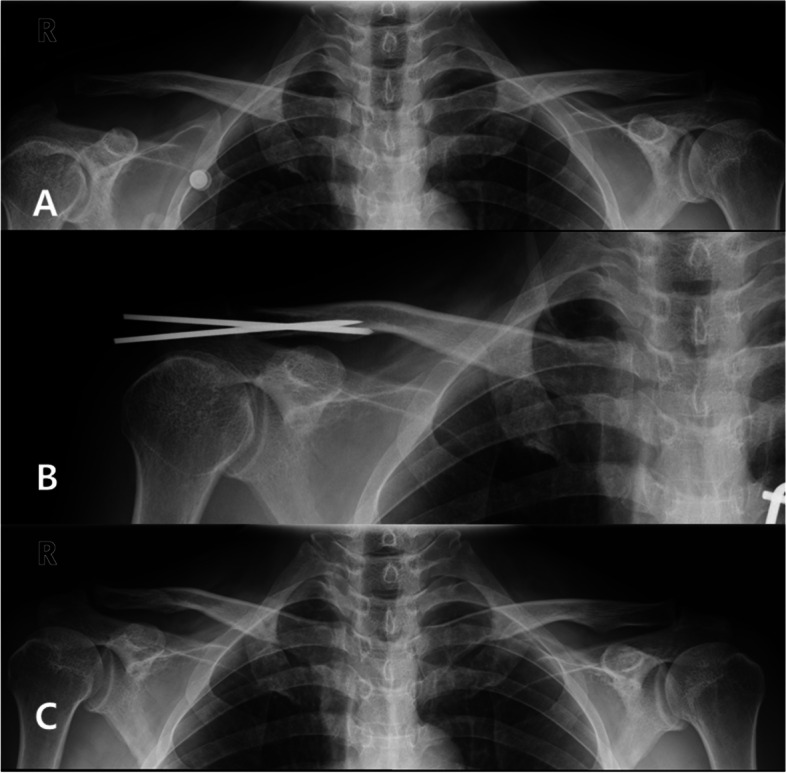
**a** Type V AC dislocation by Rockwood classification. **b** AC joint reduction and reconstruction of CC ligament with PLMT, and S-pins fixation. **c** The CC and AC distances were well maintained in the final follow-up X-ray

The mean CC and AC distances in the uninjured shoulder were 6.92 ± 1.82 (range, 3.65–9.96) mm and 3.48 ± 1.17 mm (range, 1.1–6.44 mm) respectively. The mean preoperative CC and AC distances in the injured shoulder were 16.49 ± 3.73 mm (range, 8.5–26.4 mm) and 13.84 ± 3.98 mm (range, 6.62–23.11 mm) respectively. The CC distances were 7.16 ± 1.22 mm (range, 3.85–13.23 mm) at 6 weeks after surgery and 9.29 ± 2.72 mm (range, 4.54–15.3 mm) at the final follow-up. The AC distances were 3.86 ± 2.34 mm (range, 1.56–7.13 mm) at 6 weeks after surgery and 5.30 ± 2.09 mm (range, 1.1–10.92 mm) at the final follow-up (Fig. 3). Statistical analyses were applied to the radiologic measurements to compare preoperative and postoperative data. No significant correlations were found between ASES/CS and radiologic outcomes at the final follow-up. Compared with the preoperative measurements, the mean CC and AC distances remained decreased at the final follow-up and these differences were statistically significant (*P* < .001) (Table 3).

**Table 3:** Summary of radiologic outcomes

Radiologic parameters	Results
CC distance, mm	
Uninjured	6.92 ± 1.82 (3.65–9.96)
Preoperative, injured	16.49 ± 3.73 (8.5–26.4)
Postoperative, injured	7.16 ± 1.22 (3.85–13.23)
Last follow-up	9.29 ± 2.72 (4.54–15.3)
Difference (injured – uninjured)	9.57 ± 3.49 (3.72–21.5)
Difference (preop. – postop.)	7.24 ± 3.39 (0.67–19.3)
*P* value: preop. vs. last follow-up	< 0.001*
*P* value: uninjured vs. last follow-up	0.032*
AC distance, mm	
Uninjured	3.48 ± 1.17 (1.1–6.44)
Preoperative, injured	13.84 ± 3.98 (6.62–23.11)
Postoperative, injured	3.86 ± 2.34 (1.1–7.13)
Last follow-up, injured	5.30 ± 2.09 (1.1–10.92)
Difference (injured – uninjured)	9.96 ± 3.90 (2.57–19.29)
Difference (preop. – postop.)	8.13 ± 3.46 (1.11–17.97)
*P* value: preop. vs. last follow-up	< 0.001*
*P* value: uninjured vs. last follow-up	0.025*

Values are reported as mean ± standard deviation

*CC* Coracoclavicular, *AC* Acromioclavicular

*Independent paired t-test

### Complications

**Fig. 4:**
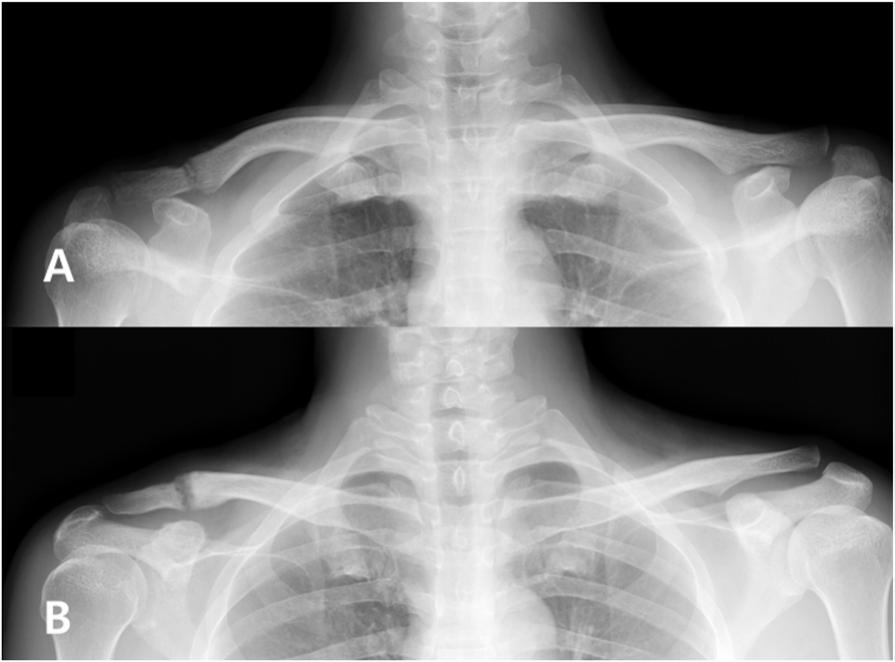
A patient (in 40’s) underwent CC reconstruction. **a** After 5 months, the patient fell down and developed a clavicle fracture at the site of the CC ligament reconstruction area. **b** After conservative treatment with a Kenny-Howard brace, union was achieved without further displacement

**Fig. 5:**
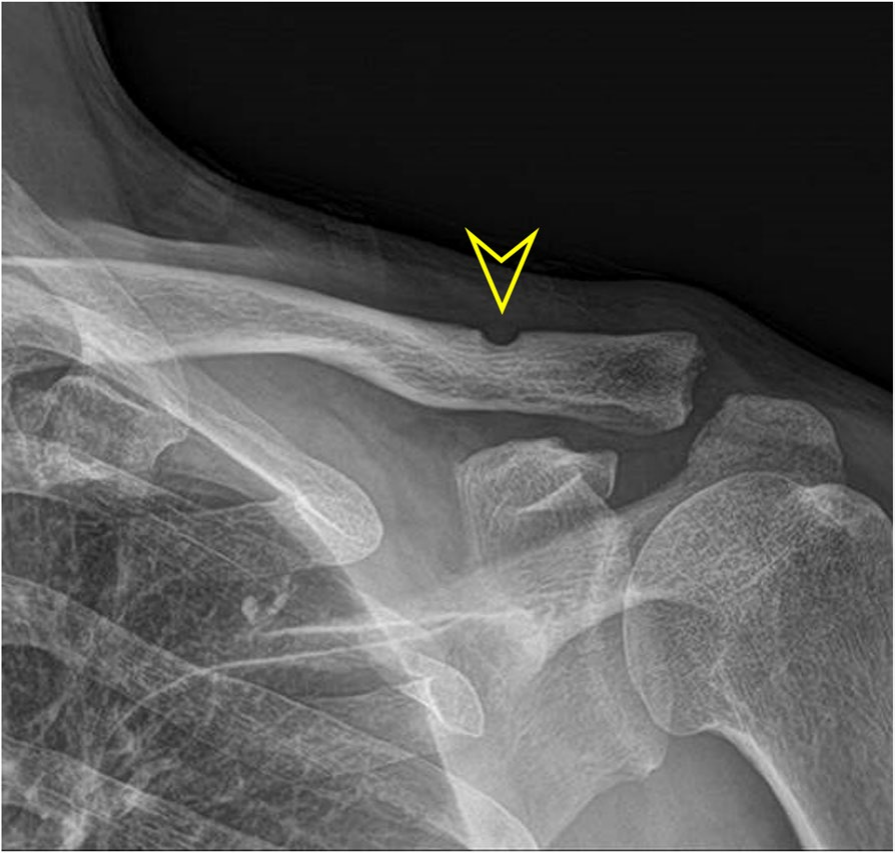
A plain radiograph taken at postoperative 18 months shows erosion of the superior cortex of the clavicle (arrow head). However, the CC and AC distances are well maintained

A slight re-widening of the CC distance occurred in 10 patients (13.1%), of which 7 patients were chronic cases (more than 6 weeks from the trauma). Two patients had clavicle fractures at the reconstructed ligament area after a simple fall down. One patient was treated conservatively (Fig. 4) with velpeau sling for 5 weeks, and bone union was obtained 3 months after the trauma. The other patient underwent open reduction and plate fixation because there was a displacement at the fracture site. There were 10 patients with pin site related complications. Three patients with pin site infection were treated using oral antibiotics. Migration of the S-pins before 6 weeks occurred in 7 patients. To stop further re-widening, 3 patients additionally wore the Kenny-Howard brace, and patients wore the velpeau brace. X-rays were checked every week, and if re-widening did not progress, additional immobilization was stopped and rehabilitation treatment was re-started. A variable amount of erosion of the superior cortex of the clavicle was detected in 69 cases (90.7%). The erosion phenomenon was regarded as the result of continuous load to the clavicle by the grafted PLMT, and interpreted as a sign that the reconstructed ligament had remained in a functional status (Fig. 5). The cortical erosion of the two fall-down patients with clavicle fracture was 2.26 and 0.75 mm (Table 4).

**Table 4:** Complications

Variable	Data
Re-widening of CC distance ^a^	10 (13.15%)
Pin site problem	10 (13.16%)
Superficial infection	3
Pin migration	7
Fracture of clavicle after slip down	2 (2.6%)
cortical erosion of clavicle	2.26/0.75 mm
Heterotopic ossification	10 (13.5%)
Cortical erosion of clavicle	69 (90.79%)
< 1 mm	26
1–2 mm	41
> 2 mm	2

Data are reported as numbers of patients and percentage

^a^Increase of CC distance over 50% compared to the uninjured shoulder

## Discussion

The various treatments for AC joint dislocation have been as follows: (1) reduction of the AC joint with simple pinning (Phemister technique) or hook plate fixation, (2) CC screw fixation (Bosworth technique), (3) direct repair of the AC or CC ligament, (4) distal clavicle resection, (5) dynamic muscle transfer, and (6) reconstruction of the CC ligament with free tendon graft [5, 10]. While, no consensus has been achieved on the best surgical method for AC joint dislocation, a number of procedures have been introduced according to these concepts. However, reconstruction of the CC ligament has been reported to have favorable results, and was discussed in many recent studies for the treatment of AC joint dislocations. Different surgical methods using various materials have been introduced. The main issues are as follows: (1) which materials will be used, artificial tapes or autologous tendons, (2) single or double bundle, (3) and whether to use bone tunnels or not [9, 11, 17, 29]. Which method to choose among these factors and combinations of techniques will depend on the experience and judgment of the operating surgeons. In our study, we used the autologous ipsilateral PL tendon with Mersilene tape in a single bundle and did not use a bone tunnel in the coracoid process and clavicle. We cannot prove that PLMT is superior to auto-graft. This technique was used with a tendon that can be harvested from the ipsilateral upper extremity, and used together with Mersilene tape to overcome the weakness of thickness and strength of PL. The technique for encircling the clavicle and coracoid process with PLMT was used under the assumption that the tendon could be fixed and maintained without making bone tunnels.

Regardless of the materials used, reducing the dislocated AC joint accurately and performing anatomic reconstruction of the CC ligament are important to achieve successful clinical and radiologic results. When using the autologous tendons, surgeons usually harvest tendons from the leg, such as the gracilis, semitendinosus, or peroneus longus tendons rather than from the arm, probably due to the advantages in diameter, length, and strength, related overall to the graft [22, 28, 29]. The PL tendon is rarely used for the treatment of AC dislocations because it is considered relatively weak for a single graft and it is also absent in approximately 10–15% of humans [20]. Due to these shortcomings of the PL tendon, surgeons usually prefer to use the autologous tendons from the leg. However, if surgeons use the PL tendon, preparing the lower extremity for tendon harvesting is not necessary as well as a functional deficit is not apparent at the wrist and forearm after harvesting the PL tendon. Thus, it can be a good candidate for graft material. To overcome the limitations of the PL tendon, we reinforced the tendon by interweaving it with artificial tape. If confirmed that the PL was absent before surgery, we would have used a tendon from the leg. However, there weren’t any patients without a PL tendon in all cases.

The healing process of autografts has four stages after applications: necrosis, revascularization, cellular proliferation, and remodeling [2]. After incorporation, grafted tendons lose their original strength up to 30–40% [2, 9]. Because grafted tendons are initially weak during stages of necrosis and revascularization, failure of single autologous tendon graft for acute AC joint dislocations was reported by Choi et al. [5] Some studies reported good results after reconstruction with artificial tapes [7, 27, 30], but artificial tapes or suture materials have no biological properties and revascularization process, thus the tapes may eventually rupture from repetitive loads [23]. For optimal outcomes, grafting materials should not only have initial strength but should also allow continuous biologic tissue ingrowth to resist continuous and repetitive load. So, we devised a technique using the autologous tendon interweaved with artificial tape, and this stabilization of AC dislocation using the PLMT offered advantages. Reconstruction with PLMT managed to overcome the disadvantages of each material and maximized the advantages. Until the grafted tendons gain sufficient strength, the artificial materials serve to add additional resistance. The rate of re-widening of the dislocated AC joint in this study was 13.1%. Compared with recent meta-analysis studies [11, 13, 22], our series showed better results in maintaining reduction and functional gains. Some surgeons perform a double loop technique instead of a single loop to increase the strength of grafts [3, 21]. Since PLMT has two graft materials, although it is a single strand, it can act as a double loop.

We did not fully dissect the detotrapezial fascia and muscles, but dissection was performed to allow passing the PLMT underneath the coracoid process. Less soft tissue dissection can help for indirect healing in acute cases. We tried to perform dissection or periosteal stripping as minimal as possible. It would be ideal if good treatment results were obtained with a minimal procedure, but even within the Rockwood classification, the degree of damage may vary and the healing potential of ligament is different for each patients. In addition, although S-pin fixation for AC joint and immobilization for 6 weeks has a very important effect on initial strength for AC-CC injury, we experienced patients whose CC distance gradually increased after the removal of S-pins. Therefore, we think that reconstruction with PLMT has the advantage of reinforcing the soft tissue healing and ensuring better treatment results.

In the reconstruction of the CC ligament for AC dislocations, some surgeons prefer making holes in the clavicle or coracoid process to pass the grafting materials. Since the introduction of double-tunnel reconstruction of the CC ligament by Mazzocca et al. [15] several studies have reported high success rates from this technique [3, 4, 12, 21]. However, as the graft material transfers axial load to the clavicle, stress fracture may occur at the weak point of the clavicle, whether the graft material is single or double [14, 16, 25]. To prevent these undesirable fractures, we passed the PLMT under the coracoid and tied it over the clavicle without drilling holes or bone tunnels through the clavicle and coracoid process. This technique has the advantages of reducing operation time, decreasing the possibilities of stress fractures, and preventing tendon rupture around the bone holes. Moreover, it allows unrestricted movement between the clavicle and coracoid process maintaining the interval between them.

The weight of the arm is transferred to the reconstructed ligament, and shoulder motion and grafted tendon ac as a stress riser leading to erosion and indentation at the superior cortex of the clavicle. In our cases, as time went by, as the grafted tendons worked against repetitive load, erosion was noted around 8 to 12 weeks postoperatively. As the clavicle adapted to the load and PLMT became stronger through fibrosis or remodeling, erosion stopped over time. However, since the grafted PLMT may act as a long-term stress riser at the site of erosion, the possibility of fracture of the clavicle should be explained to the patients, and long-term follow-up will be needed to check additional changes at the upper cortex of the clavicle.

Among the 10 patients who experienced re-widening of the CC distance at the final follow-up, 8 patients had tolerable subjective symptoms and range of motion. Therefore, the patients were satisfied with the condition and we did not recommend reoperation. In 2 cases, patients complained of pain in the AC joint and protrusion of the distal clavicle, but refused reoperation. The re-widening of the CC distance occurred in 7 out of 15 patients in chronic cases (46.7%). Thus, chronicity was considered to be a risk factor in the failure of ligament reconstruction. If there are patients who need re-operation due to pain and disability, we recommend a re-reconstruction using auto-graft tendon or a tendon harvesting from the lower extremity. Pin migration occurred in 7 cases because of the use of smooth pins instead of threaded pins for AC joint fixation. As the possibility exists that the issue may cause serious problems around the neck, we bent the tips of the pins or used a stopper to prevent complications in recent cases.

This study has several limitations. Since it is not a comparative study, the superiority over other surgical techniques could not be demonstrated. Some of the cases had a relatively short follow-up period (24 months); thus, late complications such as clavicle fractures could not be accurately assessed. To prove the advantages of the PLMT proposed in this study, a biomechanical study is necessary to compare the strengths of the PLMT with the strengths of the tendons of lower extremities. The late pathologic changes of the reconstructed PLMT should be proven through and in vivo study. Also histological studies on the difference in cell ingrowth into the autologous tendon, artificial tape, and a combination of these two materials are also warranted. At least, since follow-up MRI was required to conform the condition of the reconstructed PLMT, it is an important limitation of this study.

## Conclusion

As the reconstruction of CC ligament with PLMT obtained good radiologic and clinical results, this technique can be another grafting alternative for the surgical treatment of AC dislocation. An artificial tape may assist in reinforcing a relatively weak and thin autologous PL tendon. The PL tendon can help the artificial tape through cellular proliferation and remodeling in the late stage of healing, eventually resulting in good reconstruction results of the CC ligament. In terms of the results, this technique is more suitable for acute cases rather than chronic cases. Although re-widening occurred frequently in chronic cases, but considering that the patient’s pain and clinical symptoms have been improved, PLMT reconstruction can be applied to the chronic cases as well.

## Data Availability

The datasets used and analyzed during the current study are available from the corresponding author on reasonable request.

## Abbreviations

AC: Acromioclavicular
CC: Coracoclavicular
PMLT: Palmaris longus tendon interweaved with Mersilene tape
VAS: Visual Analogue Scale
ASES: American Shoulder and Elbow Surgeons rating scale
CS: Constant Score
AP: Anteroposterior
